# Pulmonary cavitation in patients with thyroid cancer receiving antiangiogenic agents

**DOI:** 10.1186/s12885-020-07693-5

**Published:** 2020-12-02

**Authors:** Saumil Datar, Maria Cabanillas, Ramona Dadu, David Ost, Horiana B. Grosu

**Affiliations:** 1 Department of Internal Medicine, The University of Texas MD Anderson Cancer Center, Houston, TX USA; 2 Department of Endocrine Neoplasia and Hormonal Disorders, The University of Texas MD Anderson Cancer Center, Houston, TX USA; 3 Department of Pulmonary Medicine, The University of Texas MD Anderson Cancer Center, 1515 Holcombe Blvd, Houston, TX 77030 USA

**Keywords:** Thyroid malignancy, Antiangiogenic agent, Tyrosine kinase inhibitors

## Abstract

**Background:**

Thyroid malignancies are among the most common endocrine cancers worldwide. Owing to the angiogenic nature of these malignancies, tyrosine kinase inhibitors (TKIs) are an attractive potential treatment. However, TKIs have been associated with an increased risk of tumor cavitation, in turn linked to poor outcomes, in patients with malignancies in the lungs, where thyroid cancer commonly metastasizes.

**Method:**

We performe d a retrospective cohort study of patients with thyroid cancer and evidence of metastatic disease to the lung that were treated with multi-targeted antiangiogenic TKIs. The primary objective of this study was to determine the incidence of pulmonary cavitation. The secondary objective was to evaluate the effect of pulmonary cavitation on survival.

**Results:**

Of the 83 patients with pulmonary nodules, 10 developed cavitation during treatment. Of these 83 patients, two patients had to stop the treatment due to pneumothorax. Additionally, cavitation did not demonstrate any significant effect on survival.

**Conclusion:**

In patients with thyroid cancer and evidence of metastatic disease to the chest, the use of multi-targeted TKIs led to cavitations that were not uncommon but clinical consequences were marginal. Treatment was stopped only in two patients that developed pneumothorax, however the small sample is a strong limitation of our study.

## Background

Thyroid cancer is the most common endocrine malignancy and is the ninth most common cancer overall [1]. The formation of new blood vessels, or angiogenesis, is important for tumor growth, and vascular endothelial growth factor (VEGF) has an important role in this process [2]. Thyroid tumors are highly vascular and overexpress VEGF; thus, therapy targeting VEGF receptors (VEGFRs), by which VEGF mediates its effects on angiogenesis, was found to be promising [3]. Another approach to blocking the VEGF pathway is preventing activation of VEGFRs using tyrosine kinase inhibitors (TKIs) [2]. Recent trials with multi-targeted TKIs have shown better survival outcomes for patients with metastatic thyroid cancer [4–8].

However, patients with lung cancer treated with antiangiogenic agents commonly develop cavitation in their lung lesions—that is, a gas-filled area in the center of a lung nodule [9–11]. Cavitation of malignant tissue may be caused by treatment-related necrosis, cyst formation, or desquamation and then liquefaction of tumor cells within the lesion [12]. In 2008, Marom et al. reported that 17 of 124 (14%) patients with advanced lung cancer developed tumoral cavitation during antiangiogenic therapy using bevacizumab, vandetanib, sorafenib, erlotinib, AMG-706 (multi-targeted TKI), ADH-1 (N-cadherin inhibitor), or squalamine (VEGF inhibitor) [9]. Similarly, Crabb et al. reported marked cavitation of pulmonary lesions in eight of 33 patients (24%) treated with the VEGF inhibitor cediranib [10].

The presence of cavitation in primary lung tumor has previously been associated with a worse survival suggesting that perhaps these tumor cells have a certain characteristic that supports rapid tumor growth and disease progression [13, 14]. Lung metastases, from other primary sites, may also cavitate, but do so less frequently, in an estimated 4% of metastases, however data on thyroid metastases is limited [12].

The most common sites of distant metastases for thyroid cancers are the lung and bone [15] but the true incidence of pulmonary cavitation in patients with metastatic thyroid cancer is not known. To date, pulmonary cavitation has been reported anecdotally as an unexpected adverse event in patients with thyroid cancer. In this study, we determined the incidence and clinical characteristics of pulmonary cavitation in patients with thyroid cancer and lung metastases treated with antiangiogenic TKIs.

## Methods

### Patient selection

**Fig. 1:**
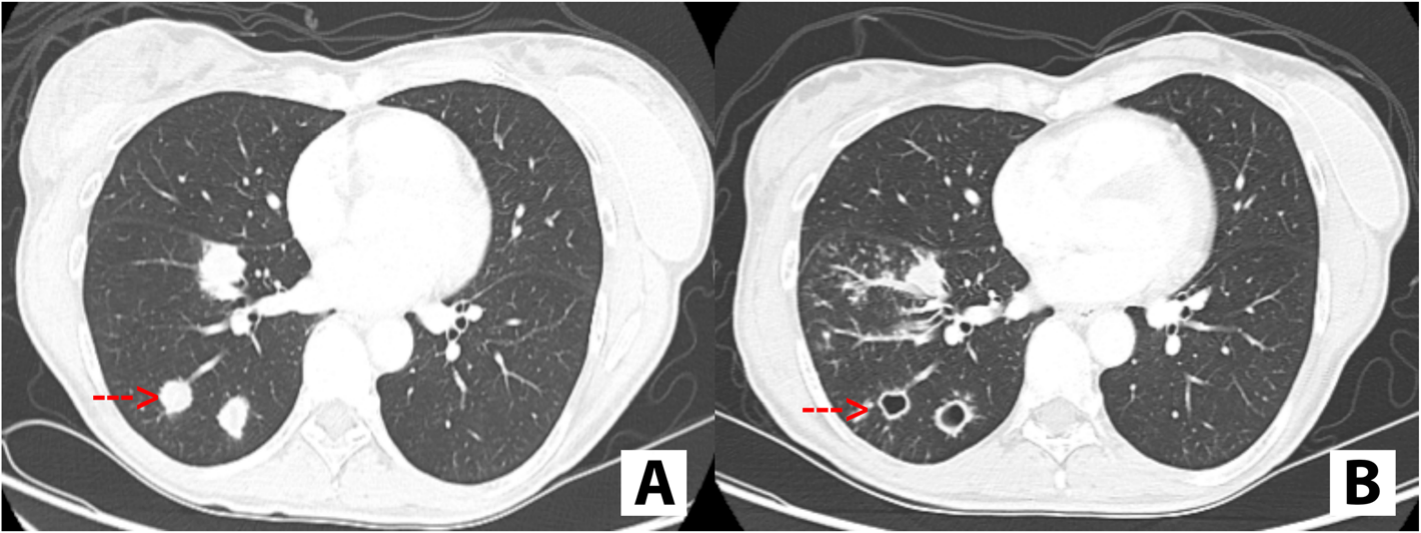
**a**. Computer tomography of the chest depicting solid pulmonary nodules, consistent with metastatic disease (red arrow). **b**. Computer tomography chest depicting cavitation in the known solid nodules (red arrow)

This retrospective cohort study was approved by the Institutional Review Board at The University of Texas MD Anderson Cancer Center. From the specialty pharmacy database and electronical medical records at our institution, all patients with thyroid cancer treated with multi-targeted antiangiogenic TKIs during January 2012 through January 2017 were selected for review. Of these, we included in our final analysis only patients with evidence of metastatic disease to the lungs, defined either as imaging, demonstrating multiple lung metastases in a typical clinical pattern, or as biopsy- or cytology-proven thyroid cancer metastatic disease. Only patients with presence of cavitation in a prior lung nodule were included. Patients in whom cavitations were felt to be due to a different reason (i.e. infection) were not included in this study. Figure 1 illustrates an example of cavitation while on treatment with TKIs.

### Endpoints

The primary objectives of this study were to determine the incidence of cavitation within lung metastases in patients with thyroid cancer treated with antiangiogenic TKIs. Secondary objective was to evaluate whether patients who develop cavitation have an overall survival disadvantage and to evaluate the clinical impact of cavity formation on additional adverse effects during therapy, including pneumothorax, as well as therapy changes or cessation.

### Statistical analysis

Mean and standard deviation were used to describe continuous variables distributed normally. Medians and interquartile ranges were used for non-normally distributed data, and frequencies were used for categorical data. For time-to-event outcomes, and Cox proportional hazard regression was used. We defined a two-tailed *P*-value of < 0.05 as statistically significant for all analyses. Intercooled Stata 13 was used for the analysis.

## Results

Eighty-three patients had evidence of thyroid cancer metastases to the lungs during treatment with antiangiogenic TKIs. Ten patients (12%) developed pulmonary nodule cavitation during treatment, and in one of those patients, the treatment was stopped because of cavitation and the resulting pneumothorax. In another patient the treatment was stopped because of pneumothorax in the absence of cavitation. Most of the patients had papillary carcinoma, followed by follicular and poorly differentiated carcinomas. Medullary thyroid carcinoma was very rare in our cohort. Treatment was not stopped or changed in any of the other patients unless there was evidence of disease progression. Patients with papillary thyroid cancer and those with pleural based lesions had a statistically significant trend towards a higher rates of cavitation. Patients’ clinical characteristics are detailed in Table 1.

**Table 1:** Characteristics of patients with metastatic thyroid cancer to the lung treated with TKI by cavitation status

Covariate	Entire cohort (***N*** = 83,%)	Cavitation (***N*** = 10,%)	No cavitation (***N*** = 73,%)	***P***-value
**Male, no.**	39 (47%)	4 (40%)	35 (47.9)	0.637
**Age, mean ± standard deviation**	60.9 ± 13.4 years	64.9 ± 6.5 years	60.39 ± 14.0 years	0.317
**Race/ethnicity, no.**				0.313
White	50 (60%)	5 (50%)	45 (61.6%)	
Black	8 (9.6%)	0 (0.0%)	8 (11%)	
Hispanic	17 (20.5%)	3 (30%)	14 (19%)	
Asian	7 (8.4%)	2 (20%)	5 (6.8%)	
**Cancer type, no.**				0.068
Papillary	39 (47%)	7 (70%)	32 (43.8%)	
Follicular	6 (7.2%)	2 (20%)	4 (5.4%)	
Poorly differentiated/anaplastic	26 (31.3%)	1 (10%)	25 (34.2%)	
Other	12 (14.4%)	0 (0.0%)	12 (16.4%)	
**Pleural base lesions on imaging, no.**	15 (18%)	4 (40%)	11 (15%)	0.055
**Emphysema on imaging, no.**	6 (7.2%)	0 (0.0%)	6 (8.2%)	0.347
**Prior radiation to the chest, no.**	2 (2.4%)	0 (0.0%)	2 (2.7%)	0.596
**Drug used, no.**				0.579
Lenvatinib	68 (82%)	7 (70%)	61 (83.5%)	
Sorafenib and lenvatinib	10 (12%)	2 (20%)	8 (11%)	
Other	5 (6%)	1 (10%)	4 (5.4%)	
**Lung metastasis responded to TKI, no.**	37 (44.5%)	6 (60%)	31 (42.4%)	0.295

*TKI* tyrosine kinase inhibitor

The incidence rate of developing cavitation while on TKI was 0.0001281 per person days with a total person days of 78,085 days.

The incidence rate of developing pneumothorax in patients that developed cavitation was 0.0000962 per person days with a total person days of 10,397, while the incidence rate of developing pneumothorax in patients that did not developed cavitation was 0.0000146 per person with a total person days of 68,416 days. The incidence risk difference was not significant at 0.0000816, 95% CI (− 0.0001091–0.0002722, *p* = 0.2683).

The median survival time was 1027 days (9–3045). On univariate analysis, the only significant risk factor was cancer type: poorly differentiated or anaplastic carcinoma was associated with a higher risk of death (*P* < 0.001) (Table 2).

**Table 2:** Risk factors for death in patients with metastatic thyroid cancer to the lung treated with TKI

Covariate	Univariate model			
	Hazard ratio	95% confidence interval		***P***-value
**Age**	0.988	0.966–1.01		0.345
**Male**	1.149	0.615–2.147		0.662
**Cancer type**				
Papillary	Ref.			
Follicular	1.741	.0489–6.191		0.391
Poorly differentiated/anaplastic	5.276	2.451–11.354		**< 0.0001**
Other	1.134	0.432–2.974		0.797
**Pleural base lesions on imaging**	0.802	0.353–1.819		0.598
**Emphysema on imaging**	0.768	0.235–2.512		0.663
**Prior radiation to the chest**	1.221	0.166–8.971		0.844
**Cavitation**	0.808	0.312–2.089		0.661

## Discussion

**Fig. 2:**
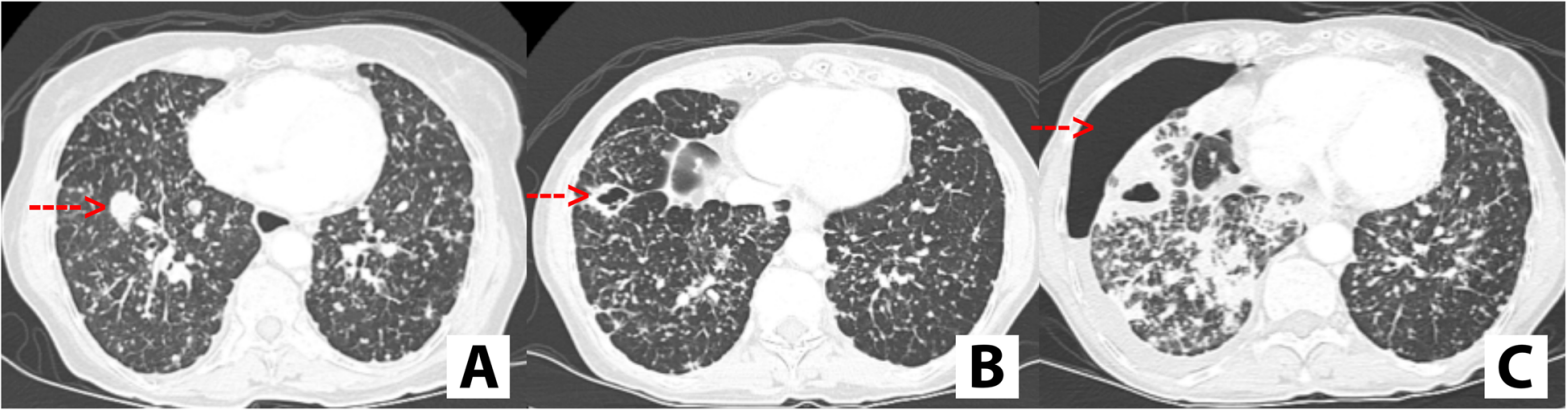
**a**. Computer tomography showing innumerable pulmonary nodules with red arrow pointing to a larger solid nodule in the right lower lobe. **b**. Cavitation of the larger solid nodule in the right lower lobe (red arrow) **c**. Computer tomography depicting development of pneumothorax (red arrow)

In 83 patients with lung metastases from thyroid cancer treated on multi-targeted antiangiogenic TKIs, the prevalence and impact of pulmonary cavitation was not uncommon at 12%, but the clinical consequences were marginal. Only two patients developed pneumothorax while on treatment with TKI and the incidence risk difference of developing pneumothorax was not significant in those patient that developed cavitation and those who did not develop cavitation while on TKI. Figure 2 illustrates a patient on TKI that developed pneumothorax.

The multi-targeted antiagiogenic TKIs lenvatinib, sorafenib, vandetanib, and cabozantinib each target multiple pathways and are under investigation for use in various thyroid cancer subtypes. Lenvatinib is being investigated for treatment of differentiated thyroid cancer and is a multi-targeted TKI of the VEGFR1-VEGFR3, fibroblast growth factor receptor 1 through 4 (FGFR1-FGFR4), platelet-derived growth factor receptor α (PDGFRα), the rearranged during transfection (RET) proto-oncogene, and v-kit Hardy-Zuckerman 4 feline sarcoma viral oncogene homolog signaling networks [4]. The drug is under investigation for treatment of anaplastic thyroid cancer. Sorafenib is also approved for differentiated thyroid cancer and is an oral dual-action inhibitor of Raf-kinase and multiple tyrosine kinases including VEGFR2, VEGFR3, PDGFR, and c-kit [2]. These two targeted agents were by far the most common ones used in our cohort. Vandetanib and cabozantinib have both demonstrated clinical activity in medullary thyroid cancer. Vandetanib is an inhibitor of the RET kinase, VEGFR, and epidermal growth factor receptor signaling and [16]. cabozantinib is a TKI of the hepatocyte growth factor (MET) receptor, VEGFR2, and RET [17].

A few studies in patients with lung cancer have reported a rate of cavitation as high as 24% with antiangiogenic agents [10]. If these agents indeed increase the risk of pulmonary cavitation, this risk would be problematic in patients with metastatic thyroid cancer, as the lung is the most common site of distant metastases and antiangiogenic TKIs are frequently used for to treat thyroid cancer. It has been suggested that all antiangiogenics have the potential to induce pulmonary nodule necrosis and cavitation, increasing risk of rupture and pneumothorax [18]. Antiangiogenic agents that target VEGFRs could cause cavitation of lung lesions and increase risk of pneumothorax [9, 10, 19]. However, among our cases, only 10 (12%) patients had evidence of cavitation, and only two developed pneumothorax, leading to drug discontinuation. We could speculate the thyroid cancer itself, not the antiangiogenic agents, increases the risk of cavitation and pneumothorax however in view of lack of comparators, such as patients with metastatic thyroid cancer who are not on TKIs we can’t be sure. Furthermore, the cavitation that did occur in our cohort had no significant effect on survival.

Our study has limitations inherent to retrospective analyses, which are subject to selection bias. In addition, the small sample size may be explain the lack of difference we observed between patients with lesions that cavitate and those without cavitation, in addition we don’t have a meaningful comparison with other subgroups, e.g., according to specific drug used or according to cancer type, or patients who were not on TKI and had metastatic disease to the lung.

## Conclusion

In patients with thyroid cancer and evidence of metastatic disease to the chest, the use of multi-targeted TKIs led to cavitations that were not uncommon but clinical consequences were marginal. Treatment was stopped only in two patients that developed pneumothorax, however the small sample is a strong limitation of our study.

## Data Availability

The datasets used and/or analyzed during the current study are available from the corresponding author on reasonable request.

## Abbreviations

VEGF: Vascular endothelial growth factor
VEGFR: VEGF receptors
TKI: Tyrosine kinase inhibitors
FGFR1-FGFR4: Fibroblast growth factor receptor 1 through 4
PDGFRα: Platelet-derived growth factor receptor α
RET: Rearranged during transfection
